# Inflammatory and Non-Inflammatory Mechanisms Controlling Cirrhosis Development

**DOI:** 10.3390/cancers13205045

**Published:** 2021-10-09

**Authors:** Paula Sánchez Sánchez, María del Mar Rigual, Nabil Djouder

**Affiliations:** Molecular Oncology Programme, Growth Factors, Nutrients and Cancer Group, Centro Nacional de Investigaciones Oncológicas, CNIO, ES-28029 Madrid, Spain; psanchezs@cnio.es (P.S.S.); mrigual@cnio.es (M.M.R.)

**Keywords:** cirrhosis, hepatic progenitor cells, hepatic stellate cells, myofibroblasts, regenerative nodules, fibrosis, extracellular matrix, inflammation, immune factors

## Abstract

**Simple Summary:**

The liver is continuously exposed to several harmful factors, subsequently activating sophisticated mechanisms set-up in order to repair and regenerate the damaged liver and hence to prevent its failure. When the injury becomes chronic, the regenerative response becomes perpetual and goes awry, leading to cirrhosis with a fatal liver dysfunction. Cirrhosis is a well-known risk factor for hepatocellular carcinoma (HCC), the most common, usually lethal, human primary liver neoplasm with very limited therapeutic options. Considering the pivotal role of immune factors in the development of cirrhosis, here we review and discuss the inflammatory pathways and components implicated in the development of cirrhosis. A better understanding of these circuits would help the design of novel strategies to prevent and treat cirrhosis and HCC, two lethal diseases.

**Abstract:**

Because the liver is considered to be one of the most important metabolic organs in the body, it is continuously exposed to damaging environmental agents. Upon damage, several complex cellular and molecular mechanisms in charge of liver recovery and regeneration are activated to prevent the failure of the organ. When liver injury becomes chronic, the regenerative response goes awry and impairs the liver function, consequently leading to cirrhosis, a liver disorder that can cause patient death. Cirrhosis has a disrupted liver architecture and zonation, along with the presence of fibrosis and parenchymal nodules, known as regenerative nodules (RNs). Inflammatory cues contribute to the cirrhotic process in response to chronic damaging agents. Cirrhosis can progress to HCC, the most common and one of the most lethal liver cancers with unmet medical needs. Considering the essential role of inflammatory pathways in the development of cirrhosis, further understanding of the relationship between immune cells and the activation of RNs and fibrosis would guide the design of innovative therapeutic strategies to ameliorate the survival of cirrhotic and HCC patients. In this review, we will summarize the inflammatory mechanisms implicated in the development of cirrhosis.

## 1. Introduction

The liver is one of the most important metabolic organs in the body, which has the impressive potential to regenerate in order to maintain tissue homeostasis after injury or partial resection of the organ. Due to the continuous exposure of the liver to different external stress signals, including toxins, alcohol abuse, hepatitis viral infections, nutrient surpluses, autoimmune diseases or genetic and metabolic disorders among others [1], which may potentially induce cell death and injury, complex wound-healing and regenerative processes are activated to repair the damaged organ, avoiding its failure [2]. Not only can these external factors activate recovery mechanisms in the liver but, additionally, gut-derived endotoxemia can drive an inflammatory response in the organ as a consequence of the release of the gut-derived bacterial endotoxin, called lipopolysaccharide (LPS), whose levels are elevated in two important chronic liver diseases, non-alcoholic fatty liver disease (NAFLD) and alcoholic-liver disease (ALD) [3].

**Figure 1:**
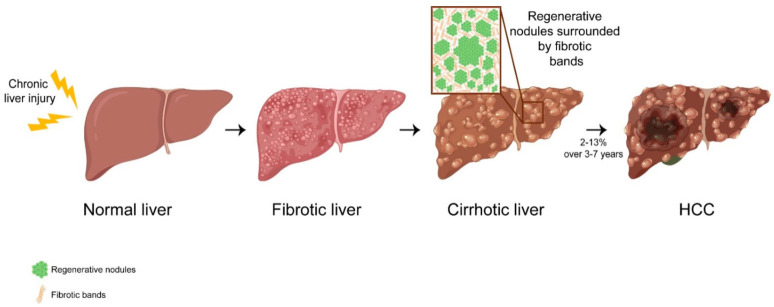
The chronic liver injury leads to cirrhosis and consists of the activation of a complex regenerative response that involves two different processes: (i) the activation and expansion of HPCs leading to the formation of the regenerative nodules and (ii) the activation of HSCs that transdifferentiate into myofibroblasts which secrete and deposit several components of ECM, forming fibrotic bands. The continuous and awry activation of both cellular processes leads to liver cirrhosis. Around 2–13% of cirrhotic patients finally develop HCC. This image was created with BioRender (https://biorender.com/ accessed on 10 September 2021).

Depending on the duration (acute/chronic) and the severity of the liver damage, the regenerative response involves different cell types and signaling pathways. Under homeostatic conditions or when the liver injury is acute, hepatocytes are the cell of origin of renewed liver tissue, with minimal contribution of the hepatic progenitor cells (HPCs), also known as oval cells, ductular cells or cholangiocytes in mice and liver progenitor cells in humans [2,4,5,6,7]. However, when injury becomes chronic and hepatocyte proliferation is completely blocked by the damage, the regeneration goes askew, leading to a sophisticated regenerative response implicating two main processes: (i) the expansion of HPCs that leads to the formation of the RNs [4,8] and (ii) the activation of the quiescent liver fibroblasts known as hepatic stellate cells (HSCs) that become myofibroblasts responsible for the deposition of the extracellular matrix (ECM), promoting fibrosis [9]. The continuous activation of these two cellular processes leads to liver cirrhosis (Figure 1). Histologically, cirrhosis is characterized by the distortion of the liver parenchyma composed of RNs and surrounded by dense and vascularized fibrotic septa, linking portal tracts with central veins and each other. Hepatic vascular architecture thus becomes completely altered, causing portal hypertension and liver dysfunctions [10,11,12].

### 1.1. HPC Expansion and Formation of RNs

In the last years, it has been well accepted that HPCs constitute a subpopulation of cholangiocytes, resident epithelial cells with progenitor and stem cell-like properties, located around the canal of Hering or intrahepatic bile ductules [13]. Under homeostasis, these progenitor cells have insignificant proliferative capacity. However, when liver injury becomes chronic, HPCs acquire the capacity to proliferate in the so-called “oval cell expansion” or “ductular reaction” to regenerate the damaged liver [4,5,6,14,15,16,17,18,19,20]. The discovery of shared mutations between HPCs and RNs, and lineage tracing experiments in a model of hepatocarcinogenesis, indicate that HPCs give rise to RNs, the main pathological feature of cirrhotic livers. RNs are defined as parenchymal and visible structures forming small benign tumors surrounded by fibrotic tissue and which protrude from the liver. RNs are formed by hepatocytes derived from HPCs that are entrapped by the deposition of extracellular matrix components produced by HSCs [4,8].

### 1.2. Activation of HSCs and ECM Production

HSCs are non-parenchymal liver fibroblasts localized between hepatocytes and sinusoids in the perisinusoidal space, known as the space of Disse. Under homeostasis, HSCs are quiescent cells implicated in the storage of vitamin A or retinoid in lipid droplets [21]. Following liver injury and different signals, quiescent HSCs become activated, converting into proliferative cells called myofibroblasts. Myofibroblasts, characterized by alpha-smooth muscle actin (α-SMA) expression, produce inflammatory and fibrogenic mediators responsible for types I and III collagen deposition which can lead to the accumulation of ECM within the injured liver environment, resulting in the promotion of fibrogenesis or fibrosis [22]. When the damage is acute, excessive ECM is usually resolved and degraded by different enzymes (e.g., the metalloproteases MMP2 and MMP14) [23,24]. When injury becomes chronic, ECM is permanently synthesized, promoting stiffness, mechanical forces and affecting liver architecture and function [22].

### 1.3. Cirrhosis and Hepatocellular Carcinoma

According to the results from the Global Burden of Diseases, Injuries and Risk Fac-tors Study (GBD), around 2.4% of total deaths in the world in 2017, representing for up to 45% of all deaths in the Western countries, were caused by liver cirrhosis, which has produced more than 1.32 million deaths globally. Over the past few years, the number of clinical trials of potential antifibrotic therapies has been increased and even some of them based on several drugs, such as obethicolic acid (OCA), selonsertib (SEL) or elafibranor, are now in phase II and III. In spite of these advances, responses are very modest and most of the anti-fibrotic therapies failed. Currently, the most effective and important treatment for liver fibrosis remains on the prevention and attenuation of the causative agent of the disease [25,26]. Dramatically, cirrhosis can also progress to HCC, the most common, usually lethal, human primary liver neoplasm, considered to be the second cause of all cancer-related deaths and the fourth most frequent cancer worldwide (GLOBOCAN 2018), with very limited, virtually inexistent, therapeutic options [27]. It is well known that around 90% of HCC cases occur in a background of chronic liver disease [28]. Although there are several studies demonstrating that NAFLD-related HCC cases can occur in the absence of cirrhosis, the percentage of these patients is still not completely clear and fluctuates among different studies, therefore cirrhosis is considered to be the strongest risk factor for this type of cancer [29,30,31].

At early stages, when the tumor is solitary and its burden is low, liver resection and transplantation are the most common procedures. However, liver transplantation is limited due to the scarcity of the donors. For patients who are not appropriate candidates for tumor resection or transplantation, several ablation techniques are indicated, including percutaneous radiofrequency ablation, microwave ablation, cryoablation and ethanol injection. When the tumor progresses to intermediate stages, transarterial treatments, such as transarterial chemoembolization (TACE) and selective internal radiation therapy (SIRT), are considered to be better treatments. Alternatively, for patients with a far-advanced HCC stage, systemic therapies are employed, even in combination with transarterial treatments. Until now, some systemic drugs have been approved by the Food and Drug Administration (FDA) for frontline advanced HCC treatment, such as Sorafenib and Lenvatinib, and for second-line therapy, such as Regorafenib [28]. In addition, different ongoing clinical trials testing immunotherapy as another possible therapeutic option have already shown some promising but narrow results. The treatment of HCC patients with cytotoxic T-lymphocyte-associated protein 4 (CTLA-4) inhibitor tremelimumab or with programmed cell death 1 (PD-1) immune checkpoint inhibitors nivolumab and pembrolizumab, produced a response in some patients, improving their survival [28]. Nonetheless, more clinical trials and research are required to establish immune-based therapies, alone or in combination with other procedures, for HCC treatment. Understanding cellular and molecular mechanisms of cirrhosis development will help to find new therapeutic strategies to prevent and cure HCC.

## 2. Mechanism-Based Immune Responses

Severe liver injuries could trigger various immune reactions, also leading to the activation of resident macrophages (Kupffer cells) and the migration and infiltration of bone-marrow derived macrophages (BMDMs), to participate in the wound healing process [2]. The inflammation response involves the secretion of chemokines, cytokines and other factors [32,33] responsible for the activation, proliferation, migration and differentiation of HPCs and/or HSCs [34].

### 2.1. Immune Factors in the Activation of HPCs

**Figure 2:**
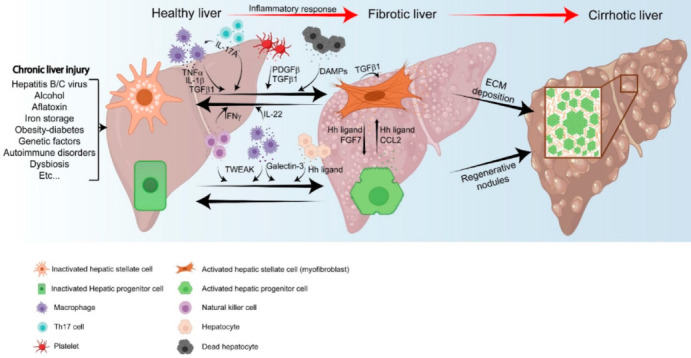
Different immune factors are responsible for the activation of HPCs and HSCs during the cirrhotic process. (i) Macrophages secrete TWEAK and IFNγ that activate HPCs and HSCs, respectively. (ii) Damaged and dysfunctional hepatocytes are responsible for the secretion of galectin-3 and Hh ligands involved in HPC expansion. Moreover, dead hepatocytes secrete DAMPS that can act directly on HSCs leading to their activation. (iii) Several cytokines activate HSCs, including TNFα, IL1β, TGFβ1, IL22 (macrophages), IL-17A (Th17 cells), TGFβ1, PDGFβ (platelets) and IFNγ (NK). (iv) HSCs could control HPC expansion by the secretion of FGF7 and Hh ligands. Vice versa, HPCs could regulate HSC activation through CCL2 and Hh ligands. (vi) HSCs secrete TGFβ1 leading to their own activation. The activation of both cell types leads to the formation of regenerative nodules and deposition of extracellular matrix, part of the cirrhotic process. This image was created with BioRender (https://biorender.com/ accessed on 10 September 2021).

One of the first and better studied growth factors involved in the activation of HPCs is the tumor necrosis factor-like weak inducer of apoptosis (TWEAK), a member of the TNF-α family. In 2005, Jakubowski and collaborators demonstrated that the overexpression of TWEAK in mice induced HPC proliferation [35], whereas its deletion inhibited their expansion after diethoxycarbonyl-1,4-dihydrocollidine (DDC) treatment [35]. Later, it was demonstrated in mice fed with a choline-deficient ethionine-supplemented diet (CDE), a model of chronic liver injury, that TWEAK is produced and secreted by natural killer (NK) cells and macrophages to activate HPC proliferation. HPCs reportedly express the TWEAK receptor Fn14 and TWEAK induces oval cell expansion in a Fn14 dependent manner via NF-kB activation [36]. Another study showed that a single infusion of bone-marrow derived cells (BMDCs) into undamaged livers was sufficient to induce oval cell response, which was inhibited by the deletion of Fn14. This suggests that TWEAK produced by macrophages is responsible for the expansion of HPCs (Figure 2), excluding the role of other factors generated during chronic liver injury, such as matrix remodeling [37].

The important role of macrophages in HPC activation is also reflected by the study of Ishikawa and collaborators [38], who demonstrated that deletion of c-Met in liver epithelial and stromal cells by using Alb-Cre and Mx1-Cre, respectively, decreases the levels of stromal derived factor-1 (SDF-1), a well-known chemoattractant for BMDMs [39]. Consequently, recruitment of BMDMs to the liver as well as levels of the metalloproteinase MMP9, implicated in the ECM degradation, are reduced [39], thereby affecting the balance between ECM production and degradation, leading to structural abnormalities that inhibit the proliferative, migration and differentiation capacity of HPCs [38]. Yet, the reduction in the number of HPCs observed by Ishikawa and colleagues could also be directly attributed to the decrease of SDF-1, independently of the parenchymal structural changes. Indeed, during chronic liver damage, SDF-1 expression is reportedly upregulated in rat livers that could directly act and activate HPCs, which express C-X-C motif chemokine receptor 4 (CXCR4), the SDF-1 receptor [40]. In support of this, some studies have shown that HSCs can secrete SDF-1 [41,42], which plays an essential role in the recruitment of myeloid-derived suppressor cells in the liver, consequently promoting HCC [42]. Therefore, considering these studies, decreasing SDF-1 or its production by HSCs in the model described by Ishikawa and collaborators could reduce HPC proliferation in a direct way and by indirect structural changes due to an impaired recruitment of BMDMs.

Macrophages could also play a role in the lineage specification of HPCs. During extensive hepatocellular damage, macrophages infiltrate into the liver and, after engulfing hepatocyte debris, express high levels of canonical ligand Wnt3a, leading to the activation of the Wnt pathway. Consequently, the activation of Wnt signaling leads to the differentiation of HPCs into hepatocytes [43].

Dysfunctional hepatocytes can also induce the ductular reaction and/or maintain the fate of HPCs by secreting several physiological and metabolic signals, such as galectin-3, α-ketoglutarate and Hedgehog (Hh) ligands [4,43,44] (Figure 2). Tummala and collaborators showed that transformed hepatocytes produce two paracrine signals, α-ketoglutarate and galectin-3, involved in the preservation of the HPC undifferentiated state and in the maintenance of HPC stemness, expansion and aggressiveness, respectively [4]. Moreover, galectin-3 is highly expressed in mouse and human hepatocytes during chronic liver injury, [45] and is upregulated in HCC patients, correlating with poor prognosis. Galectin-3 is also expressed on macrophages [45] and, in agreement with this, mice deficient in galectin-3 show less proliferative HPCs, reduced macrophage infiltration and an atypical laminin sheath around HPCs during chronic hepatocellular and biliary damages. Additionally, loss of galectin-3 reportedly leads to a block of HPCs in the G0/G1 phase and a decrease in the activation of the downstream integrin signaling intermediates. Galectin-3 has thus been proposed to modulate the adhesion to laminin and the consequent activation of integrin signaling, leading to the activation of genes responsible for cell cycle progression [46]. In line with these findings, chemical inhibition of galectin action leads to a reduction of HPC expansion [4].

Hh ligands are secreted by dysfunctional hepatocytes. Jung and collaborators showed that inhibition of hepatocyte proliferation by specific deletion of inhibitor kappa B kinase (IkkB) induces the secretion of Hh ligands into the microenvironment that act on HPC, promoting their growth and proliferation [47], highlighting the tuned crosstalk between damaged hepatocytes and HPC proliferation.

### 2.2. Activation of HSCs

#### 2.2.1. Role of Immune Factors

Kupffer cells and BMDMs are reportedly involved in the synthesis and secretion of the transforming growth factor (TGF)-β1 (Figure 2), a multifactorial cytokine and key regulator of the conversion of HSCs to myofibroblasts [48]. Cai and collaborators identified the chemokine CXCL6 which acts and activates TGF-β1 secretion from macrophages leading to the activation of HSCs [48,49]. TGF-β1 binds and phosphorylates the TGF-β1 type I receptor to activate the SMAD transcription factors to promote the expression of collagen expression type I, ECM genes [50,51] as well as α-SMA expression [52]. Interestingly, HSCs could also secrete TGF-β1, perpetuating their activation via a positive feedback loop [53] (Figure 2). TGFβ1 activates downstream signaling pathways such as mitogen-activated protein kinases (MAPKs) and, in turn, c-Jun N-terminal kinase (JNK) to induce the synthesis of platelet derived growth factor (PDGF), TGFβ1 and angiotensin II, thereby triggering the development of the contractile, proliferative and migratory phenotype of myofibroblasts in a long-lasting circuit [54,55], continuously generating fibrotic bands.

TNFα is also released by Kupffer cells to activate HSCs (Figure 2). TNFα downregulates BMP and activin membrane-bound inhibitor (BAMBI) [56], which contributes to the enhancement of TGF-β signaling in HSCs during liver fibrosis. TNFα also upregulates tissue inhibitors of metalloproteinase-1 (TIMP1), preventing HSC from ECM degradation and apoptosis [55,57,58].

Inflammasome is another key modulator of liver fibrosis, creating an inflammatory environment which triggers HSC activation [59,60]. Inflammasome controls the activation of caspase 1 and the release of IL-1β [60], an important profibrogenic signal, stimulating HSCs via the downregulation of BAMBI [61]. Additionally, the constitutive activation of NLRP3 inflammasome with a hyperproduction of IL-1β leads to spontaneous liver injury and fibrosis [62]. Inhibition of IL-1α and IL-1β in vivo protects mice from non-alcoholic steatohepatitis [63,64]. In line with these findings, IL-1β increases IL-17A secretion by Th17 cells, thereby activating HSCs [65]. IL-17A production by Th17 cells is also critical for the development of NASH [66,67,68] and HCC in mice [68,69]. Genetic ablation or chemical inhibition of the IL-17A axis in mice is sufficient to prevent diet-induced obesity [68], fibrosis and HCC development [69]. IL-17A also induces the production of IL-6, IL-1β, CCL2, TNFα and TGFβ1 in Kupffer cells and HSCs via NF-kB and STAT3 activation [32,55,70,71], enhancing the fibrotic process. Inhibition of IL-17A or suppression of Th17 differentiation can be therapeutically manipulated to reduce the fibrotic process. Digoxin, a cardiac glycoside and inhibitor of the transcription factor RORγt, critical for Th17 cell differentiation or IL-17A blocking, can be a valuable agent to prevent and treat fibrosis [68].

Bile acids have been demonstrated to induce HSCs proliferation [72]. They are also elevated in patients with liver diseases [73]. Conversely, recent studies demonstrate their anti-inflammatory role via the suppression of NF-κB-dependent signaling pathways and inhibition of the NLRP3-dependent inflammasome activities [74,75,76]. In addition, some secondary bile acids, such as 3-oxoLCA and isoalloLCA, can suppress Th17 cell differentiation and enhance Treg cell differentiation, respectively, regulating the inflammation [74,75,76]. Thus, considering these new studies, bile acids could play an anti-fibrotic role by inhibiting Th17 cell differentiation, leading to a decrease of IL-17A and, consequently, a reduction of HSC proliferation and fibrosis.

IFNγ secreted by NK cells could act on HSCs to abolish the fibrogenic response [48,77] (Figure 2). IFNγ suppresses proliferation, α-SMA expression and downregulates TGFβ1 [78,79]. IFNγ can induce death of activated HSCs via TRIAL and FasL associated death domains [78]. Moreover, IL-22 produced by innate immune system cells activates STAT3 signaling in HSCs which downregulates TGF-β1/NOTCH signaling, leading to senescence and death of HSCs in a STAT3-p53-p21 manner [80,81]. Conversely, IL-22 was identified as a profibrogenic cytokine by enhancing non-SMAD TGFβ1/p38 MAPK signaling in HSCs [82].

#### 2.2.2. Role of Non-Immune Cells

Platelets, hepatocytes and liver sinusoid endothelial cells (LSECs) are also described to activate HSCs. PDGFβ and TGF-β1 are critical mitogens released by platelets [83,84] (Figure 2). HSCs express high levels of the PDGF receptor, potently stimulating HSC proliferation and migration [22,85]. In support of this, depletion of platelets in Abcb4-/- mice reduces fibrosis [86].

There is also evidence that supports the fact that hepatocyte death or stress could lead to the activation of HSCs. Following damage, hepatocytes release damage-associated molecular patterns (DAMPs), profibrogenic molecules which can directly stimulate HSCs through TLR9 receptor [87,88,89] (Figure 2). Moreover, fibroblastic cells reportedly detect the apoptotic bodies released by dead hepatocytes, triggering the upregulation of TGF-β1 and the secretion of collagen type I [90,91]. Likewise, ballooning hepatocytes detected in several liver diseases, including NASH and cirrhosis, increase Sonic Hedgehog (SHH), consequently promoting fibrosis [92]. Furthermore, activation of transcriptional coactivator with PDZ-binding motif (TAZ) and notch signaling in hepatocytes, induces the secretion of Indian Hedgehog (IHH) ligands and osteopontin, leading to the activation of HSCs [93,94,95]. Although it is still not clear if IHH and SHH activate HSCs independently of TGFβ1, it has been proposed that TGFβ1 signaling can be amplified by these molecules [48,52]. Therefore, it is tempting to speculate that strategies based on the dual inhibition of TGF-β1 and IHH or SHH could be used to prevent and treat fibrosis. IL-33 secreted by hepatocytes could also promote the expansion of innate lymphoid cells in liver fibrosis and contributes to the secretion of IL-13 to activate HSC transdifferentiation [96]. In support of this, IL-33 is reportedly increased in cirrhotic patients [96].

It is also important to highlight the role of Hippo signaling pathway in the activation of HSCs. Mannaerts and collaborators described that HSCs had high levels of nuclear YAP during the early phases of their activation. Deletion of YAP in HSCs in vitro or inhibition of YAP in vivo reduced fibrosis [97]. Interestingly, under chronic liver injury, levels of YAP/TAZ are increased in hepatocytes, leading to the secretion of Cyr61 that can act as a chemoattractant of macrophages, which, in turn, are responsible for HSC activation [98].

LSECs contribute to proliferation of HSCs in injured parenchyma through the release of vascular endothelial growth factor (VEGF) [23]. Once HSCs are activated, they can also directly promote hepatic inflammation, mobilizing immune cells by secreting cytokines such as IL-6, IL-1β, TNFα and IFNγ, among others [99], or indirectly by activating T cells to secrete inflammatory cytokines such as IL-17A [100,101]. HSCs could also suppress hepatic inflammation and mediate tolerance by directly secreting inhibitory cytokines or by indirectly regulating immune cells. IL-10 secreted by HSCs has potent immunosuppressive activity through the activation of the p53 and p21 pathways [102]. CXC3CL1 is mainly expressed in HSCs and its interaction with Kupffer cells partly induces an anti-inflammatory phenotype [103,104]. Furthermore, NK cells are activated by the release of retinoic acid from activated HSCs [105]. HSCs can also activate myeloid derived suppressor cells (MSDC), which inhibits T cell proliferation [102].

Clearly, further studies are required to elucidate the role of stroma cells in the activation of HSCs and the development of fibrosis. Moreover, elucidating the pro- and anti-inflammatory properties in the fibrotic response could provide novel therapeutic strategies for cirrhosis.

## 3. The Crosstalk between HPCs and HSCs

The activation of HPCs by HSCs via collagen or growth factor production during the fibrotic process has been well documented [85,106]. During chronic liver damage, Thy1+ cells, a heterogeneous mesenchymal cell subpopulation composed of HSCs and myofibroblasts, appear around the HPC niche to activate their proliferation through the secretion of the FGF growth factor FGF7. HPC expansion after DDC treatment or bile duct ligation was almost completely suppressed in mice deficient for FGF7. In addition, the receptor of FGF7, FGF-binding protein 1 (FGFBP1), is almost exclusively expressed in HPCs [107]. Thus, FGF7 acts directly on HPCs via FGFBP1 activation, promoting their proliferation [107] (Figure 2). Besides controlling the proliferative capacity of HPC population, myofibroblasts can also modulate their cell fate through the secretion of the ligand Jagged1 that activates the NOTCH signaling pathway promoting the differentiation of HPCs into biliary epithelial cells [43].

Moreover, myofibroblasts can regulate HPC proliferation by secreting the disintegrin and metalloproteinase with thrombospondin motif 7 (ADAMTS7) which, in turn, controls the availability of connective tissue growth factor (CTGF) [108]. CTGF has been implicated in the activation and proliferation of HPCs via the TGFβ axis and integrin αvβ6. Deficiency and neutralization of CTGF and αvβ6 inhibits HPC expansion [109]. Thus, the secretion of ADAMTS7 by HSCs leads to the proteolysis and degradation of CTGF, avoiding the proliferation of HPCs [108].

Lymphotoxin beta (LT-β) could be another mediator in the crosstalk between HSCs and HPCs. Akursht and collaborators demonstrated that deletion of the LT-β receptor (LT-βR) inhibits HPC expansion in response to CDE-induced chronic liver injury [110]. In addition, LT-β and LT-βR are highly expressed in HPCs and HSCs in response to chronic liver injury, and LT-β is reportedly localized around HPCs immediately adjacent to activated HPCs in the periportal region [111], suggesting a crosstalk between HPCs and HSCs through LT-β and LT-βR pathways [111].

Another mechanism by which HSCs can control the activation and viability of HPC population during a fibrotic episode is the production of Hh ligands leading to the activation of Hh signaling pathway [44,112,113] (Figure 2). In bile duct-ligated mice, accumulation of Hh ligands, Hh receptors and transcriptional target genes have been detected in HSCs and HPCs. Co-culture between fibroblastic cells and HPCs activates the Hh pathway and, consequently, the growth of both cell types. Neutralization of Hh ligands inhibits these effects [112]. Moreover, patients with primary biliary fibrosis show high expression of Hh ligands and Hh-regulated transcription factors in HPCs [113]. Therefore, HSCs have the ability to regulate the proliferation of HPCs and HPCs can also control their neighboring fibroblastic cells through paracrine signals [112].

The interaction between HPCs and HSCs is not well characterized but some studies have shown that the secretion of CCL2 by cholangiocytes can lead to HSC activation [114,115] (Figure 2). Clearly, more work is required to better understand the complex relationship between HPCs and HSCs during chronic liver injury.

## 4. Conclusions

Clearly, inflammation is a key regulator of hepatic wound healing, fibrogenic response and cirrhosis development. However, there are still several doubts and unsolved questions that need to be addressed in the upcoming years. More work is indeed required to decipher the pro- and anti-inflammatory roles of HPCs and HSCs during the regenerative response, how this could impact on HCC development. Moreover, understanding how HPCs influence the activation and state of HSCs, and vice versa, remains to be determined. A better comprehension of these sophisticated cellular networks during liver regeneration is needed to develop novel, efficient and more specific innovative strategies to treat patients with cirrhosis, and eventually with HCC, two important deadly liver disorders.
